# Health Tract, No. 36

**Published:** 1862-02

**Authors:** 


### HEALTH TRACT, No. 36.

### NOTHING BUT A COLD.

(*From Hall's Journal of Health*, $1 *a year*, *New-York*.)

THE immediate cause of a vast number of cases of disease and death is a "cold;" it is that which fires a magazine of human ills; it is the spark to gunpowder. It was to a cold taken on a raw December day, that the great Washington owed his death. It was a common cold, aggravated by the injudicious advice of a friend which ushered in the final illness of Washington Irving. Almost any reader can trace the death of some dear friend to a "little cold."

The chief causes of cold are two: 1st, cooling off too soon after exercise; 2d, getting thoroughly chilled while in a state of rest without having been overheated; this latter originates dangerous pleurisies, fatal pneumonias (inflammation of the lungs,) and deadly fevers of the typhoid type.

Persons in vigorous health do not take cold easily; they can do with impunity what would be fatal to the feeble and infirm. Dyspeptic persons take cold readily, but they are not aware of it, because its force does not fall on the lungs, but on the liver through the skin, giving sick-headache; and close questioning will soon develop the fact of some unusual bodily effort, followed by cooling off rapidly.

A person wakes up some sunny morning, and feels as if he had been "pounded in a bag;" every joint is stiff, every muscle sore and a single step can not be taken without difficulty or actual pain. Reflection will bring out some unwonted exercise, and a subsequent cooling off before knowing it—as working in the garden in the spring-time; showing new servants "how to do," by turning themselves into chambermaids, waiters at table, and pastrycooks, Bridget being 'cute enough not to learn, "on purpose," (why should she, when she is paid full wages to oversee her mistress!) in going a "shopping," the particular pest of city husbands—an expedition which taxes the mind and body to the utmost; the particular shade of a ribbon, the larger or smaller size of a "figure" on a calico dress, or a camel's hair shawl; whether the main flower of a bonnet shall be "Jimpson" or a rose-bud; whether the jewelry shall sport a Cupid's arrow or a snake's head; these and similar debatable points on a thousand "little nothings," rouse their minds to a pitch of interest and excitement scarcely exceled by that of counselors of state in determining the boundaries of empires or the fate of nations.

Of course they went out upon that expedition dressed within an inch of life, as if for a ball, an opera, or a court reception, to return home exhausted in body, depressed in mind, and thoroughly heated; the first thing done is to toss down a glass of water to cool off the inner —— woman; next to lay aside bonnet, shawl, and "best dress," to cool the outer; then to "blaze away at every body in general, and the poor unfortunate husband in particular, if he has not had the gumption before then, to learn to give a wide berth on such occasions, to cool the upper —— man: and lastly, to put on a cold dress, lie down on a bed in a fireless room, and fall asleep, to wake up with infinite certainty, to a bad cold, which is to confine to the chamber for days and weeks together, and not unseldom, carries them to the grave!

A little attention would avert a vast amount of human suffering in these regards. Sedentary persons, invalids, and those in feeble health, should go directly to a fire after all forms of exercise, and keep all the garments on for a few minutes; or, if in warm weather, to a closed apartment, and, if any thing, throw on an additional covering. When no appreciable moisture is found on the forehead, the out-door garments may be removed. The great rule is, cool off very slowly always after the body has in any manner been heated beyond its ordinary temperature.

